# The outcomes of patients with very small coronary artery disease treated with thin strut cobalt chromium bare metal stents: an observational study

**DOI:** 10.1186/s40064-016-3350-7

**Published:** 2016-09-27

**Authors:** Muhammad Dzafir Ismail, Wan Azman Wan Ahmad, Matthias Leschke, Matthias Waliszewski, Michael Boxberger, Imran Zainal Abidin, Ahmad Syadi Mahmood Zuhdi

**Affiliations:** 1 Department of Medicine, University of Malaya Medical Centre, 59100 Kuala Lumpur, Malaysia; 2 Klinikum Esslingen, Esslingen, Germany; 3 Medical Scientific Affairs B.Braun Vascular Systems, Berlin, Germany

**Keywords:** Bare metal stent, TLR, MACE, Very small vessel disease

## Abstract

**Background:**

Percutaneous coronary interventions (PCI) in coronary artery disease (CAD) with very small vessel diameters remains controversial and challenging. These lesions are usually more diffuse, calcified and tortuous. The usage of thin strut bare metal stents (BMS) with excellent crossing profiles in a very small caliber coronary lesions has increased the likelihood of procedural success.

**Objectives:**

This observational study assessed the 9-month clinical outcomes in an ‘all-comers’ population with very small caliber CAD after implantation of thin strut cobalt chromium BMS.

**Methods:**

Thin strut cobalt chromium BMS implantation in a priori pre-defined subgroups was investigated in a non-randomized, international, multi-center ‘all-comers’ observational study. Primary end-point was the 9-month clinically driven target lesion revascularization (TLR) rate. Secondary end-points included the 9-month major adverse cardiac event (MACE) and procedural success rates. Data collection was done using an established electronic data acquisition form with built-in plausibility checks.

**Results:**

A total of 783 patients with a mean age of 70.4 ± 12.8 years were enrolled, 205 (26.2 %) of them had vessel diameters of 2.5 mm and smaller which was defined as CAD with very small reference vessel calibers. Older age and diabetics were associated with higher incidences of very small caliber vessels. The mean reference vessel diameter in the very small vessel group was 2.05 ± 0.27 mm and mean diameter for vessels >2.5 mm was 3.41 ± 0.55 mm. Pre-dilatation was performed more often in the very small vessel patients (52.2 vs. 42.2 %; p value 0.007). There was no difference in the overall technical success rates in very small vessel disease group (97.9 vs. 97.7 %). The 9-month TLR rate was 6.3 % for the very small vessels and 3.7 % for vessels >2.5 mm (p = 0.129). The 9-month and in-hospital MACE rates in the very small vessel group and patient with vessel diameters >2.5 mm were not significantly different (13.1 vs. 9.2 %; p = 0.1265 and 5.2 vs. 3.7 %; p = 0.349) respectively.

**Conclusion:**

This study has demonstrated that the use of thin strut cobalt chromium BMS in very small vessel CAD was reasonably safe and efficacious in the context of ‘real-world’ practice.

## Background

Treatment for very small vessel atherosclerosis with revascularization procedures remains a challenge in daily clinical practice. Coronary artery bypass grafting (CABG) in this group of patients is limited by high rates of graft failure (O’Connor et al. 1996) whereas percutaneous coronary interventions (PCI) are associated with increased risks of restenosis and adverse clinical outcomes (Elezi et al. 1998). PCI with bare metal stent (BMS) as compared to plain old balloon angioplasty (POBA) in small vessel disease only revealed conflicting results and only modest superiority of BMS over POBA (Park et al. 2000; Kastrati et al. 2000; Koning et al. 2001; Agostoni et al. 2005). BMS are generally associated with a higher risk of in-stent restenosis (ISR). The advances of drug-eluting stent (DES) technology have reduced the risk of in-stent restenosis (ISR) tremendously (Morton et al. 2003; Kirtane et al. 2009; Park et al. 2003; Morice et al. 2002; Moses et al. 2003). However, the incorporation of various drugs on the DES platform lead to an effective strut thickness increase. Hence, the usage of DES in very small vessel may still pose some potential issue including flexibility, deliverability and a continued risk of late and very late stent thrombosis (Farooq et al. 2011; Pfisterer et al. 2006). ISAR-STEREO and ISAR-STEREO 2 trials have demonstrated that thinner strut devices are associated with a significant reduction of angiographic restenosis after coronary artery stenting (Kastrati et al. 2001; Pache et al. 2003). Usage of a novel thin strut cobalt-chromium BMS (Coroflex^®^ Blue) in a ‘real-world’ setting including patients with acute myocardial infarction was proven to be safe and efficacious. Event free survival in this cohort of patients at 6 months was 90.8 % and the TLR rate for ISR at 6 months was 5.5 %. Furthermore, the cumulative stent thrombosis rate of 1.6 % and the absence of late stent thrombosis within 6 months is remarkably low for an all-comer cohort (Bocksch et al. 2010).

Currently, BMS is still used in cases of large luminal diameters, acute STEMI, in patients with high bleeding risk, anticipated surgical procedures for underlying illness and in situations where financial limitations may be an issue. With the latest stent architecture developments in BMS, there is a question whether it is still practical or even safe to implant BMS in lesions with very small vessel diameters.

The definition of small vessel coronary arteries involves more than an arbitrary upper limit of lumen diameters between 2.5 and 2.75 mm. Coronary stenting in small vessels has been shown to be safe, feasible and effective in the long term (Pache et al. 2006). With the availability of 2.25 and 2.0 mm diameter stents, the term very small vessel disease was suggested to those that are amenable to PCI with a 2.25 and 2.0 mm device. Despite varying definitions in the literature, small vessel coronary artery disease (CAD) is highly prevalent. It is estimated that approximately 20–30 % of patients with symptomatic CAD have small vessel CAD (Biondi-Zoccai et al. 2007). However, coronary stenting in very small vessels (lumen diameter ≤ 2.5 mm) remain controversial and challenging.

The objective of this registry was to document the safety and efficacy of thin strut BMS in an unselected patient population in which BMS implantation was deemed reasonable by the treating physician. This observational study assessed the 9-month clinical outcomes in an ‘all comers’ population with a focus lesions with very small coronary arteries in a ‘real-world’ setting.

## Methods

Thin strut bare metal stenting in a priori defined subgroups was investigated. This was a non-randomized, international, multi-center ‘all-comers’ observational study designed to evaluate the safety and efficacy of the thin strut Coroflex^®^ Blue Neo/Ultra coronary stent system (B.Braun Vascular Systems, Berlin, Germany). Patients were prospectively enrolled in 3 Asian and 17 European centers. In France this study was approved by the “Comité Consultative sur le Traitement de I’Information en matière de Recherche dans le domaine de la Santé (CTIRS dossier no. 12.384) and the Commission Nationale de I’informatique et des Libertés (CNIL, demande d’autorisation no. 912431). In Malaysia, this study received approval from the University of Malaya Medical Centre ethics committee, the Universiti Kebangsaan Malaysia ethics committee as well as from National Medical Research and Ethics Committee. The legal requirements are different in the participating countries. Inasmuch this clinical assessment was an observational study under routine use, the conduct in Germany was regulated by the German Device law (Medizin Produkte Gesetz §23b) which did not require a formal ethics vote at the time this registry was conducted. Likewise in the Netherlands, Switzerland and Croatia, ethics votes were not required given that data collection was done in a pseudonymized manner (no patient initials, only birth years and not birth dates). Also these patients would have been treated with the device independent of the data collection. The clinical follow-up was part of the routine patient care established in each participating country. With the exception of France, two votes were mandatory for the collection of data and one for the ethical conduct of the study (please see the attached documents). The responsibility for the documentation of the local requirements was at the individual centers. The Declaration of Helsinki was respected in all countries. An informed consent text specific to the registry approved by the local ethics committees was to be signed by patients prior to their enrolment as mandated by the local ethics committees.

Patients aged ≥18 years with stable angina or objective proof of ischemia or patients with acute coronary syndrome (ACS) had to meet the requirements for PCI (Windecker et al. 2014). The decision to implant BMS solely depended on the operator preference and experience. Single or multiple vessel stenting was allowed in de novo or re-stenotic lesions. The adjunct treatment with drug coating balloons (DCB) was left to the discretion of the operator. A maximum lesion length of 28 mm and reference vessel diameters from 2.0 to 4.0 mm were permissible. Vascular access via the femoral and radial route was permitted with recommended introducer sheaths of at least 5 French in diameter. Operators could also choose direct stenting at their discretion. A standard bolus of intravenous heparin (70 IU/kg) was given in all patients and supplemented when required. Platelet aggregation inhibitor loading was initiated prior to the procedure.

**Fig. 1:**
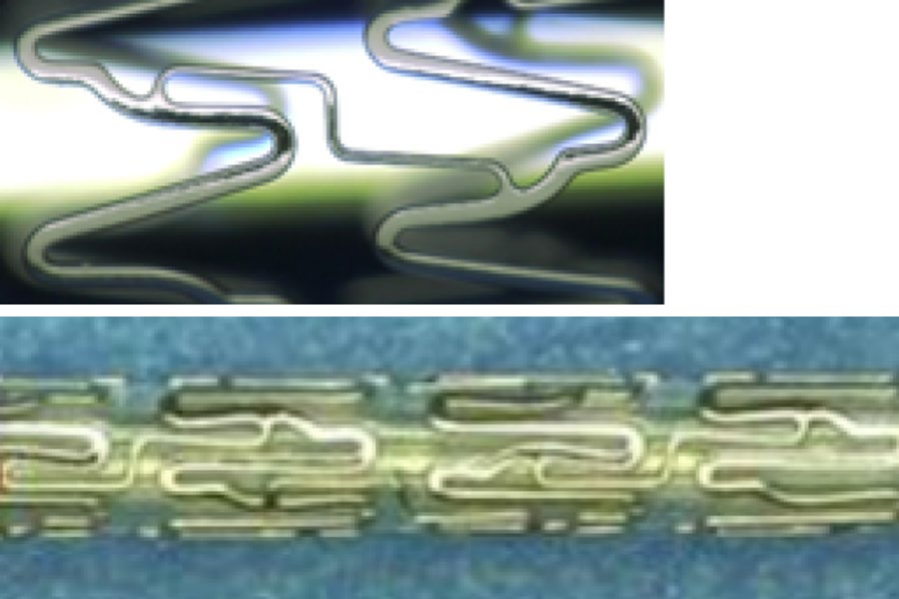
Coroflex^®^ Blue Ultra stent design (top panel). Coroflex^®^ Blue Ultra is made up of units with 6 crowns, Coroflex^®^ Blue Neo with 9 crowns, crimped stent (*bottom panel*)

The cobalt chromium Coroflex^®^ Blue Ultra/Neo is one of the latest generation coronary BMS characterized by its ultra-low strut thickness of 60 μm (Coroflex^®^ Blue Neo, vessel diameters from 2.75 to 4.0 mm) and of 50 μm (Coroflex^®^ Blue Ultra, vessel diameters from 2.0 to 2.5 mm). The stents are made of implantable, high-grade, surgical CoCr alloy (L605, ISO 5832-5 or ASTM F.90) with an established biocompatibility. This alloy has been successfully used as a material for stents and other surgical implants for many years. The stents were designed to improved deliverability in lesions that are extremely difficult to cross while reducing the metal volume in the treated vessel. The smaller caliber version is made up of units with 6 crowns whereas the larger version displays 9 crowns. Figure 1 showed the illustration of the stent. The device is available in lengths of 8–32 mm with a crossing profile of less than 0.85 mm.

DES implantation is the standard of care for most indications; however, the use of BMS still has a role in selected treatment populations particularly in some countries where the device costs are covered by the patient. Furthermore, in patients who cannot tolerate dual antiplatelet therapy (DAPT) for long periods or patients with difficult vessel anatomies. The decision to use the thin strut BMS was based on the patient’s clinical presentation and on the physician’s preferences (Windecker et al. 2014). The used investigational devices are one of the many BMS still being used in ‘real-world’ practice. The preferred BMS in this observational study has the advantage of having thin struts to allow a very favorable lesion crossability in difficult to treat lesions as compared to other less deliverable BMS.

At the discretion of the treating physician clopidogrel 75 mg/day, prasugrel 10 mg/day or ticagrelor 2 × 90 mg/day were prescribed at least 1 month post procedure and acetylsalicylic acid 100–325 mg/d for life.

In this sub group analysis we are comparing patients with very small vessel ≤2.5 mm diameter and vessel >2.5 mm diameter. From the previous literature, the definition of small vessel coronary arteries varies between 2.5 and 2.75 mm (Pache et al. 2006; Biondi-Zoccai et al. 2010). With the introduction of 2.0 and 2.25 mm diameter stents, any vessel feasible for interventions with 2.0 and 2.25 mm devices were defined as very small caliber CAD.

### Endpoints and definitions

Clinically driven target lesion revascularization (TLR) at 9-month post procedure as the composite of re-PCI or CABG was defined as the primary endpoint. Secondary endpoints included major adverse cardiac events (MACE) as the ensemble of TLR, cardiac death and myocardial infarction (MI). Rates of definite acute/sub-acute stent thrombosis and procedural success rates were also documented. MI was diagnosed with corresponding ECG changes and/or elevated troponin T or troponin I. Definite acute/sub-acute stent thrombosis were based on the ARC criteria (Cutlip et al. 2007). Cardiac death was defined as death from all causes except when the cause of death was proven non-cardiac. Procedural success was defined as achievement of less than 30 % residual in-segment percent diameter stenosis and TIMI flow 3 after the procedure using the assigned device only.

### Data collection

All data were collected through a web based data acquisition system previously used in other observational studies (Cutlip et al. 2007; Wöhrle et al. 2012; Zeymer et al. 2014). National principal investigators were responsible for the accuracy of their datasets and performed source data verification when the routinely performed web based plausibility checks indicated discrepancies.

### Statistical analysis

To prove Gaussian distribution, the Kolmogorov–Smirnov-test was used to justify the calculation of the mean and standard deviation unless otherwise indicated. Dichotomous variables were evaluated using the chi^2^-test while continuous variables were compared with the unpaired two-tailed student´s *t* test. Non-parametric test such as the Wilcoxon–Mann–Whitney rank test were used whenever applicable. For all tests the significance level α was 0.05. Based on prior result with a predecessor device (Bocksch et al. 2010), a literature value of 5.5 % for TLR was chosen. With a performance goal margin of +4.0 % and an expected 9-month TLR rate of 6.0 % a total of 490 patients would be needed to detect a difference between the null hypothesis proportion and the alternative proportion. A logistic regression model for 9-month MACE was implemented for defined cardiovascular risk factors such as presence of diabetes or ACS and lesion morphological variables such as vessel diameter, lesion length and presence of calcification. Statistical analyses were done with SPSS version 20.0 (IBM, Munich, Germany). The biometric estimate was calculated with nQuery/nTerim version 2.0 (Statistical Solutions Ltd. Cork, Ireland).

## Results

### Baseline demographics

A total of 783 patients underwent BMS implantations between 1.1.2012 and 31.12.2013. Of these patients, 205 (26.2 %) have very small vessel diameters (≤2.5 mm) and 578 (73.8 %) have vessel diameters >2.5 mm (Table 1). Patients with very small vessel diameters were older (mean age of 72.5 ± 11.3 years vs 69.7 ± 13.1 years) more diabetes (31.7 vs 22.8 %) compared to those with vessel diameters >2.5 mm. More women (24.4 vs 20.9 %; p = 0.303) and end stage renal disease patients (7.3 vs 4.0 %; p = 0.056) were in the very small vessel group. Remarkably, 316 (40.4 %) of the overall patients presented with ACS, 42.0 % in very small vessel versus 39.8 % in vessel diameters >2.5 mm. From the total of 20.8 % who had STEMI, 22.4 % with very small vessel versus 20.2 % with vessel diameters >2.5 mm.

**Table 1:** Patient demographics

Variable	All patients	Vessel diameters ≤2.50 mm	Vessel diameters >2.5 mm	p value small versus large vessel diameters
Number of patients	783	205 (26.2 %)	578 (73.8 %)	–
Number of lesions	880	247	633	–
Number of BMS used	1027	286	741	–
Age (years)	70.4 ± 12.8	72.5 ± 11.3	69.7 ± 13.1	0.008
Male gender	612 (78.2 %)	155 (75.6 %)	457 (79.1 %)	0.303
Diabetes	197 (25.2 %)	65 (31.7 %)	132 (22.8 %)	0.012
Hypertension	502 (64.1 %)	133 (64.9 %)	369 (63.8 %)	0.790
Cardiogenic shock	38 (4.9 %)	14 (6.8 %)	24 (4.2 %)	0.125
End stage renal disease	38 (4.9 %)	15 (7.3 %)	23 (4.0 %)	0.056
Atrial fibrillation	98 (12.5 %)	20 (9.8 %)	78 (13.5 %)	0.165
Mechanical heart valve	10 (1.3 %)	2 (1.0 %)	8 (1.4 %)	0.655
Documented DVT and/or PE	4 (0.5 %)	1 (0.5 %)	3 (0.5 %)	0.957
Acute coronary Syndrome (ACS)	316 (40.4 %)	86 (42.0 %)	230 (39.8 %)	0.588
STEMI	163 (20.8 %)	46 (22.4 %)	117 (20.2 %)	0.506
NSTEMI	153 (19.5 %)	40 (19.5 %)	113 (19.6 %)	0.991

*DVT* deep vein thrombosis, *PE* pulmonary embolism

### Angiographic characteristics, procedural data and co-medication

Baseline angiographic characteristics and procedural data are shown in Table 2. There was a total of 880 coronary lesions which were treated during this study. 247 (28.1 %) lesions treated were in very small vessels primarily in the left anterior descending artery (39.7 %) and left circumflex artery (37.2 %). Of note, 40.1 % of the total lesions were complex (type B2/C) according to the modified American College of Cardiology (ACC) and American Heart Association (AHA) classification (Ryan et al. 1998). Smaller vessel lesions trended to be more diffusely diseased with higher calcium burden. Pre-dilatation was performed more often in the very small vessels (52.2 %) as compared to vessel diameters >2.5 mm (42.2 %). The mean reference diameter for very small vessel group was 2.05 ± 0.27 mm; whereas the mean reference diameter for vessel diameters >2.5 mm was 3.41 ± 0.55 mm. There was no difference in terms of stent balloon inflation pressure (very small vessel 14.8 ± 3.1 atm vs. vessel diameters >2.5 mm 15.1 ± 3.0 atm) and the overall technical success rate in very small vessel group was 97.9 versus 97.7 % in the patient group with vessel diameters >2.5 mm. The new oral antiplatelet drugs prasugrel/aspirin and ticagrelor/aspirin were administered more frequently in the very small vessel (11.2 vs 9.5 %) and (7.8 vs 2.4 %) compared to vessel diameters >2.5 mm respectively (Table 3). The duration of dual antiplatelet therapy (DAPT) between the two groups were not statistically different.

**Table 2:** Lesion characteristics and procedural data

Variable	All patients	Vessel diameters ≤2.50 mm	Vessel diameters >2.5 mm	p value small versus large vessel diameters
Number of lesions	880	247	633	–
Target vessel				<0.001
LAD	290 (33.0 %)	98 (39.7 %)	192 (30.3 %)	
CX	233 (26.5 %)	92 (37.2 %)	141 (22.3 %)	
RCA	344 (39.1 %)	55 (22.3 %)	289 (45.7 %)	
graft	13 (1.5 %)	2 (0.8 %)	11 (1.7 %)	
Total occlusion	107 (12.2 %)	33 (13.4 %)	74 (11.7 %)	0.496
Chronic total occlusion	22 (2.5 %)	8 (3.2 %)	14 (2.2 %)	0.381
Thrombus burden	116 (13.2 %)	30 (12.1 %)	86 (13.6 %)	0.570
Diffuse vessel disease	442 (50.2 %)	140 (56.7 %)	302 (47.7 %)	0.017
Calcification	297 (33.8 %)	104 (42.1 %)	193 (30.5 %)	0.001
Ostial lesion	47 (5.3 %)	15 (6.1 %)	32 (5.1 %)	0.546
Bifurcation lesion	51 (5.8 %)	12 (4.9 %)	39 (6.2 %)	0.457
Severe tortuosity	104 (11.8 %)	36 (14.6 %)	68 (10.7 %)	0.114
AHA/ACC type B2/C lesion	353 (40.1 %)	104 (42.1 %)	249 (39.3 %)	0.451
Reference diameter (mm)	3.03 ± 0.53	2.05 ± 0.27	3.41 ± 0.55	<0.001
Lesion length	14.7 ± 7.8	14.7 ± 7.3	14.8 ± 8.0	0.950
Degree of stenosis (%)	86.3 ± 10.7	87.0 ± 9.7	86.0 ± 11.0	0.233
Pre-dilatation	396 (45.0 %)	129 (52.2 %)	267 (42.2 %)	0.007
BMSs used	1027	286	741	–
BMS diameter (mm)	3.02 ± 0.51	2.48 ± 0.25	3.23 ± 0.42	<0.001
BMS length (mm)	16.0 ± 5.9	15.7 ± 5.8	16.1 ± 5.9	0.399
BMS inflation pressure (atm)	15.0 ± 3.1	14.8 ± 3.1	15.1 ± 3.0	0.144
Additional DCB	17 (1.7 %)	3 (1.0 %)	14 (1.9 %)	0.344
Final result % stenosis	1.7 ± 10.0	1.7 ± 11.0	1.7 ± 9.7	0.430
Overall technical success	1004 (97.8 %)	280 (97.9 %)	724 (97.7 %)	0.849

**Table 3:** Peri-procedural drug therapy

Drug type	Drug	All patients	Vessel diameters ≤2.50 mm	Vessel diameters >2.5 mm	p value small versus large vessel diameters
Antiplatelet therapy (APT)	Aspirin	783 (100 %)	205 (100.0 %)	578 (100.0 %)	0.009
	Clopidogrel	663 (84.7 %)	164 (80.0 %)	499 (86.3 %)	
	Prasugrel	78 (10.0 %)	23 (11.2 %)	55 (9.5 %)	
	Ticagrelor	30 (3.8 %)	16 (7.8 %)	14 (2.4 %)	
	Ticlopidine	2 (0.3 %)	0 (0.0 %)	2 (0.3 %)	
	GP IIb/IIIa inhibitors	10 (1.3 %)	2 (1.0 %)	8 (1.4 %)	
Oral anti-coagulation	None	694 (88.6 %)	188 (91.7 %)	506 (87.5 %)	0.106
	Vitamin K antagonist (VKA)	74 (9.5 %)	12 (5.9 %)	62 (10.7 %)	
	New oral anticoagulation (NOAC) rivaroxaban	15 (1.9 %)	5 (2.4 %)	10 (1.7 %)	
Triple Therapy (OAC + DAPT)	OAC + Aspirin + Clopidogrel	70 (8.9 %)	14 (6.8 %)	56 (9.7 %)	0.451
	OAC + Aspirin + Prasugrel	2 (0.3 %)	0 (0.0 %)	2 (0.3 %)	
	OAC + Aspirin + Ticagrelor	1 (0.1 %)	0 (0.0 %)	1 (0.2 %)	

*OAC* oral anti-coagulation

### Clinical outcomes

**Fig. 2:**
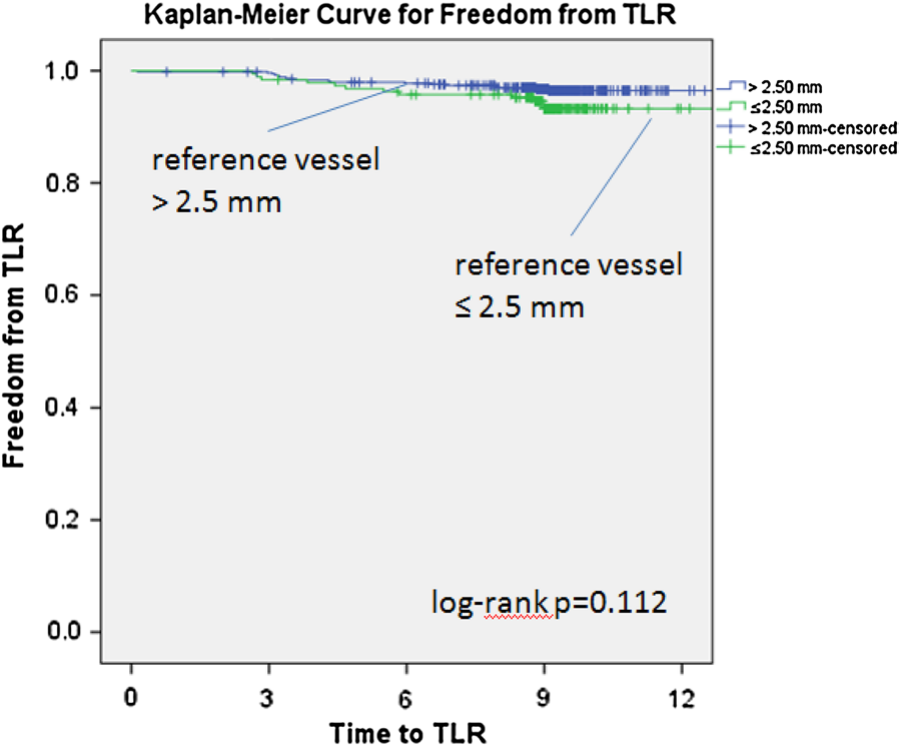
Kaplan–Meier curve for freedom from TLR in very small vessels versus larger vessels

Overall follow-up at 9 months was 93.9 %. The 9-month TLR rate was 6.3 % for very small vessels and 3.7 % for vessels >2.5 mm (p = 0.129). Figure 2 showed the Kaplan–Meier curve for freedom from TLR. The log-rank test revealed no statistical difference with p = 0.112. 9-month and in-hospital MACE between very small vessels and vessels >2.5 mm were not significantly different either (13.1 vs 9.2 %; p = 0.1265 and 5.2 vs 3.7 %; p = 0.349) respectively. There was no case of acute stent thrombosis observed in either group (Table 4).

## Discussion

Vessel size remains an important determinant of adverse outcomes even in the DES era (Togni et al. 2007). While DES are consistently less prone to restenosis, efforts are being made to narrow the efficacy gap between BMS and DES. Clinical studies have shown the potential advantage of thinner stent struts in reducing the restenosis rate (Suttorp et al. 2015). Although DES are preferred as a default treatment strategy, BMS are still implanted for reasons such as limited financial resources in some Asian countries or contraindications to prolonged DAPT. There is limited data on PCI in very small vessels involving ‘real-world’ patients. The Arthos Pico Austrian Multi-center registry recruited 203 all-comer patients with average stent diameter of 2.58 ± 0.22 mm with a device delivery success rate of 98.0 %. The patients were followed up for 6 months whereas the overall MACE rate was 13.0 % with cumulative TLR of 6.0 % (Strehblow et al. 2007) which was higher than the TLR rate in this study. In this context a word of caution is indicated when comparing clinical event rates across studies. Particularly in the case of small caliber vessels, restenosis may be asymptomatic and therefore difficult to be detected unless an angiographic follow-up is routinely conducted.

**Table 4:** Clinical outcomes

Variable	All patients	Vessel diameters ≤2.50 mm	Vessel diameters >2.5 mm	p value small versus large vessel diameters
Number of patients	783	205 (26.2 %)	578 (73.8 %)	–
Patients with clinical follow-up	735 (93.9 %)	191 (93.2 %)	544 (94.1 %)	0.627
Follow-up time (months)	9.5 ± 2.4	9.4 ± 2.1	9.6 ± 2.5	0.427
Time to discharge (days)	4.0 ± 12.8	6.0 ± 23.7	3.3 ± 4.7	0.014
In hospital MACE	30 (4.1 %)	10 (5.2 %)	20 (3.7 %)	0.349
In hospital TLR	0 (0.0 %)	0 (0.0 %)	0 (0.0 %)	–
In hospital MI	17 (2.3 %)	4 (2.1 %)	13 (2.4 %)	0.815
In hospital cardiac death	13 (1.8 %)	6 (3.1 %)	7 (1.3 %)	0.094
9-month MACE	75 (10.2 %)	25 (13.1 %)	50 (9.2 %)	0.126
9-month TLR (Re-PCI, CABG)	32 (4.4 %)	12 (6.3 %)	20 (3.7 %)	0.129
9-month CABG	5 (0.7 %)	1 (0.5 %)	4 (0.7 %)	0.759
9-month MI	38 (5.2 %)	11 (5.8 %)	27 (5.0 %)	0.669
9-month cardiac death	21 (2.9 %)	8 (4.2 %)	13 (2.4 %)	0.199
9-month stroke rate	3 (0.4 %)	1 (0.5 %)	2 (0.3 %)	0.771
9-month total definite/probable stent thrombosis	11 (1.4 %)	5 (2.6 %)	6 (1.1 %)	0.138
Acute stent thrombosis, ≤24	0 (0.0 %)	0 (0.0 %)	0 (0.0 %)	–
Subacute stent thrombosis,1-30 d	1 (0.1 %)	0 (0.0 %)	1 (0.2 %)	0.553
Late stent thrombosis, ≥30 d	10 (1.3 %)	5 (2.6 %)	5 (0.9 %)	0.081

PCI in very small vessels are often considered controversial because (1) very small vessels are responsible for the perfusion of a small coronary territory (2) they may not be worthwhile for stenting (3) they have a higher risk of dissection, perforation (4) or restenosis (5) and they are technically more challenging in terms of lesion crossability. The clinical relevance of very small vessel CAD should be interpreted within the clinical framework of the individual patient. The procedural success rate improved significantly with novel designs of newer generation BMS. In our study, we have found that the technical success in delivering the BMS into very small vessels was on the same high level with 97.9 % as compared to the patient group with vessels >2.5 mm (97.7 %). A higher percentage of very small vessel lesions received pre-dilatation, likely because of higher calcification burden and diffuse vessel disease. However, stent inflation pressures did not differ in both groups.

One of the known advantages of BMS is the duration of DAPT post PCI. The risk of stent thrombosis in DES is often related to non-compliance or premature cessation of DAPT. Hence, if compliance to DAPT becomes a concern, BMS could be a reasonable option. In this study, we have shown that BMS performed equally well when implanted in very small vessels as compared larger vessels. The implication would be shorter duration of DAPT in patients undergoing elective PCI with BMS. However, this duration may need to be prolonged in patients undergoing PCI in the setting of ACS.

Furthermore, in special populations like the Asian patients, with higher prevalence of diabetes mellitus (Ahmad et al. 2013), the elderly and females usually have diffuse and very small vessel disease. The distal parts of the artery when the artery are very small, tortuous and calcified there may be an issue with deliverability of the stent for some patients, thus new generation thin strut BMS can be considered.

### Limitations

This study has several limitations. First of all, this is a single-group, non-randomized design, which may have some degree of selection bias. The indication for BMS implantations may differ on a country basis depending on the risk profile for individual patients. Decisions may also have been impacted by local device costs in some countries. Data analysis is hence descriptive in nature and inferior to a randomized trial as no direct comparison can be made versus a control group. Secondly, the results reported here may have been affected by the type of bias inherent in all registries, namely the selective inclusion of lower-risk patients, together with less exhaustive monitoring than that applied in randomized controlled trials, potentially contributing to an overall under-reporting of events. Moreover, mandatory clinical, in-hospital follow-ups would have been preferred over telephone interviews; however, the regulatory ramification of this protocol modification would have rendered this study to be unfeasible. The follow-up rate of 93.9 % in this registry might be able to compensate for some of these deficits. Lastly the sample size calculation was based on the 9-month TLR rate of the overall population. Consequently, the descriptive nature of this subgroup analysis without the claim of proper statistical power must be mentioned as well.

## Conclusion

Ultra-thin strut cobalt chromium BMS can be used in coronary artery disease (CAD) of very small reference diameters with very a high procedural success rates. A difference in clinical event rates despite differences in cardiovascular risk factors and lesion characteristics was not observed. However, PCI with DES remains the standard of care unless cost issues have an impact on the decision making process.

## Abbreviations

ARC: Academic Research Consortium
ACC: American College of Cardiology
ACS: acute coronary syndrome
AHA: American Heart Association
BMS: bare metal stent
CABG: coronary artery bypass graft surgery
CAD: coronary artery disease
DAPT: dual anti-platelet therapy
DES: drug eluting stent
DCB: drug coated balloon
ECG: electrocardiogram
ISR: in stent restenosis
MACE: major adverse cardiac event
PCI: percutaneous coronary intervention
POBA: plain old balloon angioplasty
STEMI: ST elevation myocardial infarction
TLR: target lesion revascularization
TIMI: thrombolysis in myocardial infarction
